# 纵隔炎诊治应尽早外科干预

**DOI:** 10.3779/j.issn.1009-3419.2018.04.24

**Published:** 2018-04-20

**Authors:** 天生 闫

**Affiliations:** 北京大学第三医院胸外科 Department of thoracic surgery, Peking University Third Hospital

本期刊出的遵义医学院附院胸外科陈安平等“持续负压引流在口咽部来源急性前纵隔感染中的治疗经验”一文，对17例口咽部来源的急性下行性纵隔感染，采用胸骨后通道对接持续负压引流的方式治疗取得了很好的效果。诊断和外科引流治疗时机把握的较好，引流方式主动积极、及时充分是其可取之处。

急性纵隔炎（acute mediastinitis）是一种严重的胸外科急性重症感染性疾病，以往少见，但近年来发病有增多趋势，其发展迅速，临床经过凶险，可使纵隔内许多重要器官受累，具有较高的死亡率。病因多为继发性，常见有口咽部感染引发的下行性纵隔炎（descending necrotizing mediastinitis, DNM），以及贯通性胸部外伤、和食管气管源性感染，如食管或气管破裂、食管尖锐异物致穿孔、吻合口瘘等。

纵隔内组织结构疏松，气管、食管及大血管之间充满疏松的结缔组织及大量的淋巴组织，向上与颈部筋膜间隙相通。纵隔感染一旦发生，即可在这一封闭性通道内迅速蔓延及扩散，并伴随细菌及毒素的大量吸收，导致严重的全身感染中毒症状。发展到纵隔脓肿阶段时，过程更加急重凶险，病死率高。

典型的临床表现为起病急，有寒战、高热，胸骨后剧烈疼痛，可放射至颈部、耳后或引起耳痛。脓肿形成压迫气管可产生高音调咳嗽、呼吸困难、心动过速和发绀。严重时出现可危及生命的感染中毒性休克。查体见呼吸急促、心跳加快，有明显全身中毒症状，胸骨有压痛，颈部肿胀和扪及皮下气肿。发生于外伤、手术后的急性纵隔炎，易于诊断。有吞咽异物、颈部感染、败血症等病史发现也不困难。以颌面部、颈部及胸部高分辨增强计算机断层扫描（computed tomography, CT）检查（层厚1 mm，层间距1 mm）常有特异性的发现，可尽早明确诊断，如纵隔气肿、脓肿和液平、液气胸等征象。食管碘油造影可证实食管穿孔部位。

纵隔炎患者救治的关键是把握好治疗时机。一旦诊断明确，应尽早外科处理。治疗原则为彻底去除病因，纵隔尽早充分引流，有效控制感染，充足的营养支持。

DNM是颌面、颈部间隙感染通过颈部间隙下行扩散，侵犯纵隔及周围组织，导致的致命并发症。DNM通常是由多种微生物的混合感染引起，一旦引起纵隔感染则发展快、病情重。由于高级别抗生素的使用，典型的临床表现如高热、寒战、脓毒血症等少见，从而使临床早期诊断困难。化脓性感染沿颈间隙的迅速扩散，形成坏死性纵隔炎，可发生感染性假性动脉瘤及纵隔大血管腐蚀致突然大出血死亡。其病死率高达17%-20%。因此，延误诊断和不恰当引流是DNM威胁生命的主要原因。DNM患者多长期无法进食，且处于高代谢状态，术后常规经胃肠营养管给予肠内营养，必要时联合肠外静脉营养支持，保证充足的能量供应；针对伴有2型糖尿病患者，规律地监测血糖，使用胰岛素控制血糖。对于伴有低蛋白血症、贫血者，应及时输注白蛋白、成分血。

各种原因导致的食管、气管破裂损伤后发生的急性纵隔炎如果救治不及时，死亡率相当高。这与纵隔的解剖生理特点密切相关；纵隔感染一旦形成，炎症很快沿着疏松的结缔组织扩散且影响全部纵隔器官，临床症状极为严重；且纵隔位于躯体中心，很难作到彻底引流；病原菌又多为大肠杆菌、绿脓杆菌等，易致中毒性休克、心肺功能衰竭、大血管腐蚀性出血而出现凶险结局。如因误吞枣核、假牙落入食管等异物引起的，须取出异物并同时引流，彻底控制感染，但是，抗感染治疗只有在去除异物及进行纵隔引流后才有效。如异物已进入胸腔内，或形成一侧脓胸则须开胸取出异物同时引流。如系贯通性外伤或手术后引起的，则须根据伤情、病情进行具体处理。外科引流治疗是纵隔感染最有效的治疗方法，前纵隔感染低于第2-3肋间者作胸骨后引流；在第5胸推平面以上的纵隔感染作颈部切口引流，前述两种情况也推荐采用遵义医学院陈安平介绍的胸骨后上下对接引流的方法；第5胸推平面以下者经胸腔引流，必要时在胸腔镜或纵隔镜辅助下，以做到精确置管和充分引流；劈开胸骨手术后的前纵隔感染，应及早作胸骨后扩创引流。食管损伤性穿孔大，则需行食管修补或切除术。食管裂口的修补一般主张在发病24 h内进行，也有人认为缝合食管破裂口或切除创伤食管也不能防止炎症的扩散。此外，大量抗生素控制感染、输血、输液防治休克。支持营养、吸O_2_、物理或药物降温以减少全身消耗，均为重要措施。若为食管穿孔必须禁食，为了维持营养，可行胃或空肠造瘘术，胃肠道营养或锁骨下静脉穿刺，行深静脉营养，也都非常必要。作者经治1例肝硬化上消化道大出血急诊胃镜检查见食管破裂患者，伴有严重的低蛋白、贫血，一般状况很差。作者采用胸腔置多个引流管，充分引流，减轻和控制了感染；经胃穿刺置管入胃和十二指肠，同时作胃内引流减压和十二指肠营养；约两个月，食管破裂口生长愈合良好，痊愈出院。
